# A Monoclonal Antibody-Based Indirect Competitive ELISA for Detecting Goose Astrovirus Antibodies

**DOI:** 10.3390/vetsci13010059

**Published:** 2026-01-07

**Authors:** Junfeng Lv, Yanhan Liu, Zhihui Liu, Zhonghao Wang, Wenxuan She, Cun Liu, Ye Tian

**Affiliations:** 1 Poultry Institute, Shandong Academy of Agricultural Sciences, Jinan 250100, China; nkyljf@163.com (J.L.); 15713285323@163.com (Z.L.); xgym365@163.com (Z.W.); sssx0710@163.com (W.S.); 2 Shandong Provincial Key Laboratory of Livestock and Poultry Breeding, Jinan 250100, China; 3 Shandong Provincial Center for Animal Disease Control, Jinan 250100, China; vetlyh@163.com (Y.L.); liucun89@163.com (C.L.)

**Keywords:** goose astrovirus, VP27, monoclonal antibody, indirect competitive ELISA, serum antibody

## Abstract

Goose astrovirus (GoAstV) infection has become prevalent in major goose-producing regions, causing substantial economic losses to the industry. In this study, an indirect competitive ELISA (ic-ELISA) was developed based on a monoclonal antibody (mAb) targeting the GoAstV VP27 protein. The recombinant VP27 protein was expressed in *E. coli* and purified, followed by the generation of murine mAbs using the purified antigen. Through screening with GoAstV particles, mAb 3G11 exhibited strong immunoreactivity, which was further confirmed by Western blot and immunofluorescence assay (IFA). The ic-ELISA conditions were optimized as follows: GoAstV particle coating concentration of 10^4^ TCID_50_ per well, 3G11 mAb dilution of 1:8000, and incubation times of 120 min for coating, 60 min for serum samples, and 60 min for mAb binding. The assay exhibited satisfactory performance in terms of sensitivity, specificity, and reproducibility. Using this method, serum samples collected from major goose farming areas in Shandong province were tested and showed an overall seropositivity rate of 11.7%. This study provided a reliable serological tool for detecting GoAstV-specific antibodies and would support future vaccine evaluation efforts.

## Simple Summary

China is the world’s leading producer of geese, accounting for over 70% of global goose production. With the rapid expansion of the goose industry, the control of viral diseases has become increasingly challenging, particularly in the case of goose astrovirus (GoAstV). While vaccines against GoAstV remain in the experimental stage, comprehensive epidemiological surveillance and evaluation of immune protection are essential. In this study, we generated a monoclonal antibody (mAb) targeting VP27 using GoAstV particles to obtain antibodies with neutralizing potential. Utilizing this mAb, we established an indirect competitive ELISA (ic-ELISA) for the rapid and accurate diagnosis of GoAstV infection in goose flocks. This study provides valuable tools and insights to support future vaccine development against GoAstV.

## 1. Introduction

Goose astrovirus (GoAstV) is an emerging avian pathogen in China, having disseminated across major goose-farming regions [1,2]. Goslings infected with GoAstV develop visceral and articular gout, accompanied by anorexia, lethargy, and excretion of chalky droppings [3,4,5]. The infection induces rapid mortality in goslings, with case fatality rates of 20–50%, causing substantial economic losses to the poultry industry [6]. Although the gout-inducing mechanisms have been investigated, effective control strategies remain elusive [5,7,8].

GoAstV is a single-stranded, positive-sense RNA virus within the family Astroviridae and genus Avastrovirus [9]. Similarly to other astroviruses, GoAstV possesses an approximately 7 kb genome comprising two untranslated regions (the 5′-UTR and 3′-UTR) and three open reading frames (ORF1a, ORF1b, and ORF2) [1,10,11]. ORF1a and ORF1b encode non-structural proteins that mediate viral replication and transcription [12,13]. ORF2, encoding the structural protein, constitutes the viral capsid [14]. During host cell invasion, the ORF2-encoded polyprotein undergoes trypsin-mediated proteolytic cleavage into subunits including VP25, VP27, and VP34; notably, VP27 governs viral infectivity and serves as the dominant antigenic protein [15].

Vaccine development represents one of the most effective strategies for disease control, wherein neutralizing antibody titers serve as a critical metric for evaluating vaccine-induced immunoprotection. To date, several ELISA-based methods for detecting GoAstV-specific serum antibodies have been established, enabling diagnostic confirmation, seroepidemiological surveillance, and immune monitoring [16,17]. Furthermore, methods that could detect neutralizing antibodies should also be established to evaluate vaccine efficacy in future applications.

This study aimed to generate a murine monoclonal antibody (mAb) specific to the GoAstV VP27 protein. The immunoreactivity of the produced mAb was characterized using Western blot and immunofluorescence assay (IFA). Subsequently, a sensitive and specific indirect competitive ELISA (ic-ELISA) was developed and validated for its sensitivity, specificity, and reproducibility. This validated ic-ELISA was applied to conduct a seroepidemiological survey of GoAstV infection using clinical serum samples.

## 2. Materials and Methods

### 2.1. Cells, Virus, Sera and Animals

SP2/0 murine myeloma cells were obtained from Procell Life Science & Technology Co., Ltd. (Wuhan, China), and maintained in RPMI-1640 medium supplemented with 20% fetal bovine serum (FBS; Thermo Fisher Scientific, Waltham, MA, USA). LMH cells were preserved in our laboratory and cultured in DMEM containing 10% FBS and used for the propagation of GoAstV. One-day old geese were acquired from Guiliu Livestock Farming Co., Ltd. (Qihe, China). GoAstV-negative sera were obtained from goslings confirmed negative by PCR, while positive sera were collected at 7 days post-infection. BALB/c mice were procured from Shandong Pengyue Experimental Animal Breeding Co., Ltd. (Jinan, China).

### 2.2. Expression and Purification of VP27 Protein

Total RNA of GoAstV was extracted using the Simply P Total RNA Extraction Kit (BioFlux, Hangzhou, China). The VP27 gene segment was amplified by one-step RT-PCR using the AccurSTART One Step RT-PCR Kit (Vazyme, Nanjing, China) with the primer pair 5′-gaattcCAGGTTACTCCCTCGCT-3′ and 5′-gcggccgcAGAGGTCTTGAGCGAGAC-3′, which incorporate EcoRI and NotI restriction sites, respectively. The amplified VP27 sequence was verified by sequencing and subsequently codon-optimized for expression in *E. coli* by Sangon Biotech Co., Ltd. (Qingdao, China). The optimized VP27 gene was cloned into the pET-28a (+) vector, and the resulting recombinant plasmid, pET-28a-VP27, was confirmed by restriction digestion and DNA sequencing. The validated plasmid was then transformed into *E. coli* BL21(DE3) competent cells (TransGen, Beijing, China). Recombinant protein expression was induced by the addition of IPTG. VP27 expression was analyzed by SDS-PAGE, and the protein was purified under native conditions using His-Tagged Protein Purification Kit (CWBIO, Beijing, China). The purified VP27 was verified by SDS-PAGE and Western blot analysis using anti-His monoclonal antibody (Biodragon, Beijing, China).

### 2.3. Development of Anti-VP27 mAbs

The purified VP27 protein was emulsified with an equal volume of incomplete Freund’s adjuvant (Sigma-Aldrich, Shanghai, China). Six-week-old female BALB/c mice were subcutaneously injected in the cervical region with the emulsion containing 50 μg of VP27 protein. Immunizations were administered three times at two-week intervals. Fourteen days following the final immunization, antibody titers in serum were detected by indirect ELISA using VP27 protein as the coating antigen following procedures reported before [18]. Mice with high serum antibody titers were euthanized by cervical dislocation, and splenocytes were harvested for fusion with SP2/0 murine myeloma cells. The fused cells were cultured in HAT selection medium in 96-well plates. Cell culture supernatants were collected seven days post-fusion and screened for anti-VP27 antibodies by indirect ELISA using GoAstV as coating antigen. Hybridoma clones secreting VP27-specific antibodies were subsequently subjected to three rounds of limiting dilution to establish monoclonal lines. Monoclonal antibodies were produced as ascites by inoculating ten-week-old female BALB/c mice with the selected hybridoma cells.

### 2.4. Characterization of Anti-VP27 mAbs

The reactivity of monoclonal antibodies against the VP27 protein was assessed by Western blot analysis. Purified VP27 protein was separated by electrophoresis on a 12.5% SDS-polyacrylamide gel and subsequently transferred onto a nitrocellulose membrane. The membrane was then blocked by incubating with 5% skimmed milk for 2 h at room temperature. Following blocking, the membrane was incubated with anti-VP27 monoclonal antibodies (hybridoma supernatant) for 2 h at room temperature. After three washes with PBST, the membrane was incubated with HRP-conjugated goat anti-mouse IgG (CWBIO, Beijing, China) for 1 h at room temperature. Following a final washing step, protein bands were visualized using Amersha ImageQuant 800 (Cytiva, Shanghai, China).

An IFA was performed on GoAstV-infected LMH cells. LMH cells were seeded in 96-well plates and infected with GoAstV at a multiplicity of infection (MOI) of 0.1. At 48 h post-infection, the cells were fixed with 4% paraformaldehyde in PBS for 30 min at room temperature and stored at 4 °C until use. Fixed cells were incubated with anti-VP27 monoclonal antibodies, followed by incubation with AF488-labeled goat anti-mouse IgG (H+L) (Beyotime, Shanghai, China). DMEM and PBS were used as control for the anti-VP27 mAbs. Immunofluorescence images were acquired using a Nikon ECLIPSE Ts2R fluorescence microscope (Nikon, Tokyo, Japan).

### 2.5. Serum Sample Detection by Neutralization Test

All negative and positive serum samples used in this study were confirmed with neutralization test. Viral titer was measured with IFA using polyclonal antiserum or mAb against VP27, and calculated and expressed as TCID50. For the neutralization test, serum samples were inactivated at 56 °C for 30 min, after which 100 μL of each serum was mixed with an equal volume of GoAstV suspension containing 10^5^ TCID_50_. Following incubation at 37 °C for 1 h, the residual viral titer of each mixture was determined. Samples demonstrating a reduction in virus titer to below 10^4^ TCID_50_ were considered positive for neutralizing antibodies.

### 2.6. Development of mAb-Based ic-ELISA

GoAstV viral suspensions were diluted and coated onto 96-well plates (100 µL/well, 10^5^ TCID_50_, 10^4^ TCID_50_, 10^3^ TCID_50_) and incubated overnight at 4 °C. Following coating, the plates were washed three times with PBST and blocked with 5% non-fat milk (Beyotime, Shanghai, China) in PBS at 37 °C for 60, 90 and 120 min. After washing, 100 µL of GoAstV positive (P) and negative (N) serum (diluted 1:10 in dilution buffer) were added to the plates and incubated at 37 °C for 45, 60 and 75 min. After washing, 100 µL of anti-VP27 mAb (diluted from 1:1000 to 1:16,000 in PBST) were added to the plates and incubated at 37 °C for 45, 60 and 75 min. Subsequently, 100 µL of HRP-conjugated goat anti-mouse IgG (CWBIO, Beijing, China) was added and incubated for 50 min at 37 °C. After three additional washes with PBST, the plates were developed with TMB substrate for 15 min, and the reaction was terminated using TMB stop solution (Beyotime, Shanghai, China). Absorbance was measured at 450 nm. Optimum reaction parameters and composition were optimized based on the maximum the N/P ratio.

### 2.7. Determination of the ic-ELISA Cut-Off Percent Inhibition Value

Thirty GoAstV-negative serum samples were analyzed to establish the cut-off percent inhibition (PI) value. The PI for each sample was calculated using the following formula: PI (%) = [1 − (OD_(450)_ of sample − minimum OD_(450)_)/(maximum OD_(450)_ − minimum OD_(450)_)] × 100% [19]. The cut-off PI value was defined as the mean PI of these negative samples plus twice the standard deviation (mean + 2 × SD) [20].

### 2.8. Sensitivity, Specificity and Reproducibility Assessments of ic-ELISA

The sensitivity of the developed ELISA was evaluated using known positive serum samples subjected to serial dilutions ranging from 1:10, 1:100, 1:1000, and 1:10,000. Specificity was assessed by testing the method against antisera positive for goose Parvovirus (GPV), Tembusu virus (TMUV), avian Reovirus (ARV), avian influenza virus (AIV, H5), and duck Avastrovirus (DAstV), which were generated using vaccine strain or natural infection. To determine reproducibility, five serum samples (comprising three positive and two negative) were analyzed in triplicate, and the intra-assay coefficient of variation (CV) was calculated. The reproducibility was considered satisfactory when the CV values were below the 10% threshold [18].

### 2.9. Application of the ic-ELISA

Serum samples were collected by the Avian Pathogens Diagnosis and Monitoring Center of the Poultry Institute, Shandong Academy of Agricultural Sciences, which has obtained CNAS accreditation from commercial and breeding goose farms in Shandong province, including Weifang, Dezhou, Jining, Liaocheng, Linyi, Zaozhuang, Jinan, between October 2024 to May 2025. Samples were categorized into two types: clinical diagnostic specimens from diseased geese and epidemiological surveillance samples from healthy geese. All samples were tested using the newly established serological method.

## 3. Results

### 3.1. Preparation of VP27 Protein

**Figure 1:**
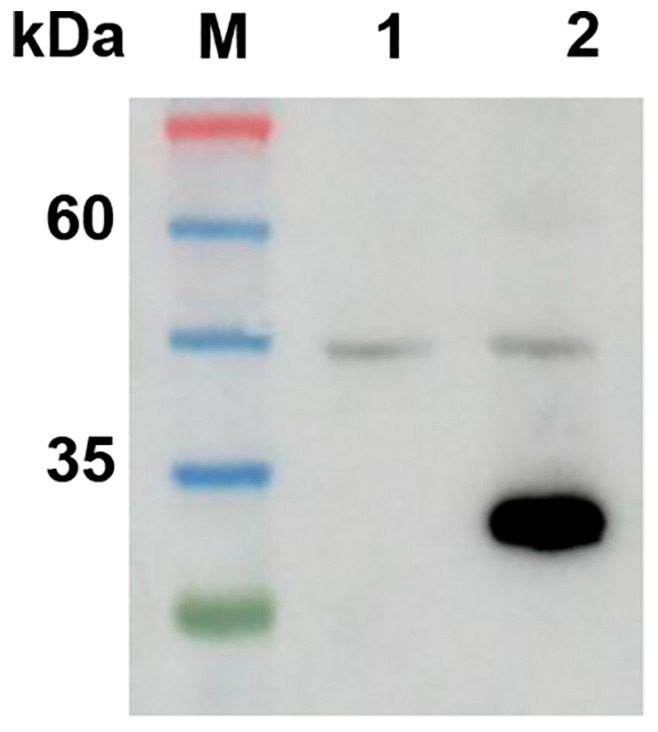
Western blot analysis of recombinant VP27 protein. Purified VP27 was probed with an anti-His tag monoclonal antibody. Lane M: protein marker; Lane 1: negative control (non-induced *E. coli* lysate); Lane 2: purified VP27 protein. Western Blot original pictures see Figure S1.

The expression of VP27 protein was tested by SDS-PAGE and confirmed by Western blot analysis, and the results showed a protein band of approximately 27 kDa, which corresponded to the expected molecular mass of VP27 (Figure 1).

### 3.2. Identification of mAbs Specific for VP27

**Figure 2:**
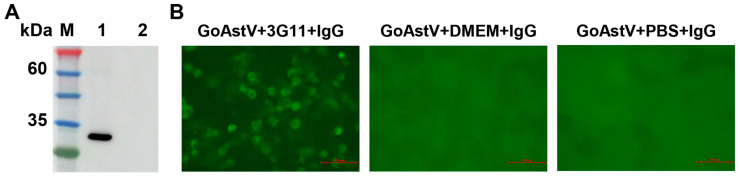
Immunoreactivity analysis of the VP27 monoclonal antibody (3G11). (**A**) Western blot analysis of mAb 3G11. Lane M: protein marker; Lanes 1 and 2: lysates of *E. coli* BL21 (pET-28a-VP27) with and without IPTG induction, respectively. (**B**) IFA of GoAstV-infected LMH cells. Cells were incubated with VP27 mAb (3G11), DMEM, or PBS, followed by staining with AF488-labeled goat anti-mouse IgG. Scale bar: 100 µm. Western Blot original pictures see Figure S1.

MAbs for VP27 were screened, and then identified based on Western blot and IFA. The results showed that hybridoma clone 3G11 could specifically recognize the VP27 protein in Western blot analysis (Figure 2A). Moreover, the antibodies effectively detected GoAstV antigens in infected LMH cells via IFA (Figure 2B).

### 3.3. Optimization of the ic-ELISA

Based on the positive-to-negative (N/P) ratio, the optimal antigen coating concentration was determined to be 10^4^ TCID_50_ per well, while the optimal dilution of the 3G11 mAbs was 1:8000 (Table 1). Following the same criterion, the incubation times for blocking, serum samples, and monoclonal antibodies were standardized at 120 min, 60 min, and 60 min, respectively (Table 2).

### 3.4. Determination of Cut-Off Value

The cut-off value for the ic-ELISA was established using thirty negative serum samples. The mean PI value and SD of these samples were determined to be 29.30% and 7.61%, respectively. Accordingly, the cut-off value was calculated as 44.52% (Table 3). Serum samples exhibiting PI values below this threshold were classified as negative, while those with values equal to or exceeding 44.52% were considered positive.

### 3.5. Sensitivity, Specificity, Reproducibility and Accuracy Assessments of ic-ELISA

All samples tested positive at dilutions of 1:10 and 1:100, while two remained positive at a 1:1000 dilution (Table 4). All samples yielded negative results at the 1:10,000 dilution (Table 4), demonstrating acceptable analytical sensitivity for this assay.

The positive antisera against GPV, TMUV, ARV, AIV H5 and DAstV were tested as negative using this method (Table 5), indicating that the method exhibits high specificity for GoAstV antibody detection.

The reproducibility of the assay was evaluated based on the coefficient of variation (CV). The intra-assay CV ranged from 3.53% to 9.82%, and the inter-assay CV varied from 3.19% to 8.42% (Table 6).

### 3.6. Epidemiological Investigation of GoAstV

A total of 196 serum samples were collected from major goose-farming regions in Shandong Province. The overall seropositivity rate for GoAstV was 11.7%, with the highest incidence (15.6%) observed in Liaocheng (Table 7).

## 4. Discussion

GoAstV infection has been identified as a primary causative agent of gout in goslings, posing a significant threat to the Chinese goose industry with substantial economic losses [21,22,23]. Consequently, the development of effective preventive and therapeutic strategies has become a research priority, underpinned by the critical need for rapid and accurate diagnostic methods [16,17]. Unlike previously characterized strains, the novel GoAstV isolate used in this study did not induce discernible cytopathic effects in available cell lines. Therefore, viral titers were quantified using an IFA in LMH cells, a system that also facilitated preliminary neutralization assessments.

The ELISA is a cornerstone serological technique in veterinary diagnostics, valued for its operational simplicity, high throughput, and robust sensitivity and specificity [16,24,25,26]. To develop an ELISA for GoAstV, we targeted the VP27 capsid protein. VP27 constitutes a major immunogenic component of astroviruses, forming the outer spikes of mature virions, mediating host cell entry, and eliciting strong immune responses [15,27,28]. Compared to the precursor VP90, VP27 presents a higher density of immunodominant epitopes [27,29]. In this study, recombinant VP27 was expressed in *E. coli* and used to generate mAbs in BALB/c mice. The resulting mAb, 3G11, demonstrated specific reactivity against both recombinant VP27 and native GoAstV particles in Western blot and immunofluorescence assays (Figure 2), confirming its suitability for serological assay development.

In present study, an ic-ELISA based on mAb 3G11 was established, in which GoAstV antibodies present in serum samples compete with mAb 3G11 for binding sites on immobilized particles. Optimal reaction conditions, including antigen concentration and antibody dilution, were determined via checkerboard titration using negative and positive control sera, as previously reported [18,24], with selection based on the highest N/P ratio (Table 1 and Table 2). A diagnostic cut-off value of 44.52% was calculated from the PI analysis of 30 negative serum samples, a threshold consistent with values reported in prior studies [16]. Following assay development, key performance parameters—sensitivity, specificity, and repeatability—were rigorously evaluated, as they are critical for reliable serological application [16,18]. The results confirmed that the assay meets the requisite standards for clinical and field use (Table 4, Table 5 and Table 6).

The development of effective vaccines is essential for controlling GoAstV infection, although no licensed vaccines are currently available [30]. Neutralizing antibody titer is a critical correlate of vaccine-induced immune protection [31]. Therefore, to enhance the probability of detecting such antibodies, intact viral particles were employed both for monoclonal antibody screening and as the coating antigen in our ic-ELISA [32,33]. The strong immunoreactivity of mAb 3G11 with viral particles (Figure 2B) suggests it may target surface epitopes, a premise requiring future validation. Consequently, the particle-based ic-ELISA is postulated to primarily detect serum neutralizing antibodies, a functionality that also necessitates confirmation through future vaccine studies.

## 5. Conclusions

In conclusion, a murine mAb (designated 3G11) was generated against the GoAstV VP27 protein. Western blot and IFA confirmed its specific immunoreactivity with both recombinant VP27 and native GoAstV particles. Subsequently, an ic-ELISA was developed using mAb 3G11, whose analytical performance in terms of sensitivity, specificity, and reproducibility was validated through comprehensive assessment. Collectively, this study establishes a reliable serological tool for detecting GoAstV-specific antibodies and provides a foundation for evaluating vaccine efficacy in future applications.

## Figures and Tables

**Table 1:** Determination of the optimal amount coating antigen and mAbs dilution.

GoAstV Coating Amount		Dilution				
		1:1000	1:2000	1:4000	1:8000	1:16,000
10^5^ TCID50	N	3.268 ± 0.715	3.020 ± 0.682	2.581 ± 0.496	2.117 ± 0.328	1.757 ± 0.306
	P	1.583 ± 0.421	1.375 ± 0.358	1.283 ± 0.314	1.105 ± 0.253	0.963 ± 0.201
	N/P	2.064	2.196	2.012	1.916	1.824
10^4^ TCID50	N	2.537 ± 0.301	2.324 ± 0.092	2.021 ± 0.102	1.752 ± 0.114	1.325 ± 0.217
	P	0.824 ± 0.098	0.619 ± 0.054	0.420 ± 0.048	0.364 ± 0.031	0.357 ± 0.017
	N/P	3.079	3.754	4.812	4.813	3.711
10^3^ TCID50	N	1.724 ± 0.257	1.452 ± 0.218	1.297 ± 0.201	1.135 ± 0.168	0.906 ± 0.153
	P	0.567 ± 0.137	0.542 ± 0.197	0.485 ± 0.108	0.385 ± 0.112	0.312 ± 0.064
	N/P	3.04	2.679	2.674	2.945	2.904

The optimal amount coating antigen and mAbs dilution was determined based on the N/P value.

**Table 2:** Determination of the optimal reaction time for ic-ELISA.

Blocking (min)	Serum Samples (min)	VP27 mAbs (min)		
		45	60	75
60	45	3.128	3.458	3.624
	60	3.215	3.687	3.765
	75	3.654	3.596	3.852
90	45	3.754	3.659	3.752
	60	3.961	3.851	4.012
	75	4.218	3.958	4.351
120	45	3.582	3.912	4.215
	60	4.372	4.621	4.635
	75	4.256	4.657	4.593

The optimal incubation time was determined based on the N/P value, and the incubation times for blocking, serum samples and VP27 mAbs were 120 min, 60 min, and 60 min, respectively.

**Table 3:** Determination of cut-off value for ic-ELISA.

Sample No.	PI	Sample No.	PI	Sample No.	PI	Sample No.	PI
1	31.70%	9	25.60%	17	30.80%	25	33.50%
2	25.30%	10	35.90%	18	34.60%	26	30.80%
3	11.20%	11	28.60%	19	10.70%	27	27.60%
4	23.50%	12	29.30%	20	24.30%	28	33.50%
5	36.40%	13	37.30%	21	33.10%	29	34.80%
6	27.80%	14	35.60%	22	34.50%	30	38.10%
7	13.10%	15	25.80%	23	35.80%		
8	35.80%	16	17.50%	24	36.40%		
Mean PI	29.30%	SD	7.61%		Cut-off value		44.52%
Cut-off value = Mean PI + 2 × SD							

**Table 4:** Sensitivity detection of ic-ELISA.

Sample No.	Dilution							
	1:10		1:100		1:1000		1:10,000	
1	65.34%	P	60.24%	P	53.20%	P	41.26%	N
2	52.51%	P	48.60%	P	42.25%	N	40.23%	N
3	72.30%	P	64.28%	P	56.31%	P	44.31%	N
4	69.54%	P	52.24%	P	43.70%	N	38.50%	N
5	60.35%	P	50.26%	P	41.25%	N	32.40%	N

Samples were all positive. P, positive. N, negative. P or N were determined based on cut-off value.

**Table 5:** Specificity detection of ic-ELISA.

	Antisera Against Different Virus											
	GoAstV		GPV		TMUV		ARV		AIV H5		DAstV	
PI	56.20%	P	34.65%	N	27.50%	N	25.40%	N	22.60%	N	35.20	N

P, positive. N, negative. P or N were determined based on cut-off value.

**Table 7:** Detection of GoAstV infection in goose farming regions in Shandong province.

Region	Serum Samples	Flock Type	Positive Samples	Positive Rate
Weifang	51	Commercial	7	13.7%
Dezhou	33	Breeding	3	9.1%
Jining	27	Commercial	3	11.1%
Liaocheng	32	Commercial	5	15.6%
Linyi	23	Commercial	3	13.0%
Zaozhuang	18	Breeding	1	5.6%
Jinan	12	Commercial	1	8.3%
Total	196		23	11.7%

**Table 6:** Repeatability (Intra- and inter-assay) detection of ic-ELISA.

	Sample	Test 1	Test 2	Test 3	Mean	SD	CV
Intra-assay	1	71.20%	68.50%	65.30%	68.33%	0.024	3.53%
	2	54.30%	60.10%	62.20%	58.87%	0.033	5.68%
	3	58.20%	65.30%	59.30%	60.93%	0.031	5.12%
	4	32.20%	27.50%	29.40%	29.70%	0.019	6.50%
	5	25.30%	30.10%	32.20%	29.20%	0.029	9.82%
Inter-assay	1	63.40%	68.70%	70.40%	67.50%	0.030	4.42%
	2	62.50%	64.10%	56.20%	60.93%	0.034	5.60%
	3	60.30%	61.20%	56.80%	59.43%	0.019	3.19%
	4	28.70%	30.20%	31.50%	30.13%	0.011	3.79%
	5	24.60%	25.20%	29.60%	26.47%	0.022	8.42%

CV, coefficient of variation. CV = SD/Mean.

## Data Availability

The original contributions presented in this study are included in the article/Supplementary Materials. Further inquiries can be directed to the corresponding author.

## Supplementary Materials

The following supporting information can be downloaded at https://www.mdpi.com/article/10.3390/vetsci13010059/s1, Figure S1: Western Blot original pictures.

## Abbreviations

The following abbreviations are used in this manuscript:

GoAstV	Goose astrovirus
ic-ELISA	indirect competitive ELISA
mAb	monoclonal antibody
IFA	immunofluorescence assay
VP	viral protein
TCID_50_	50% tissue culture infective dose
UTR	untranslated region
ORF	open reading frame
P	positive
N	negative
PI	percent inhibition
GPV	goose Parvovirus
TMUV	Tembusu virus
ARV	avian Reovirus
AIV	avian influenza virus
DAstV	duck Avastrovirus
CV	coefficient of variation
