# Supplementary figures and images for: A Monoclonal Antibody-Based Indirect Competitive ELISA for Detecting Goose Astrovirus Antibodies
Source: Vet Sci. 2026 Jan 7;13(1):59. doi: 10.3390/vetsci13010059 (PMC12846494; doi:10.3390/vetsci13010059)

Original image for figure 1

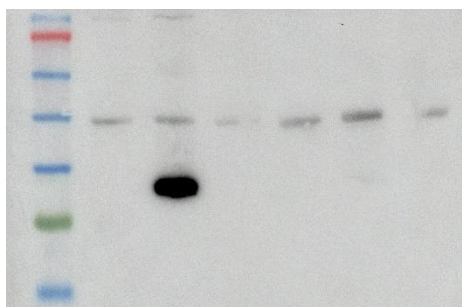

Original image for figure 2A

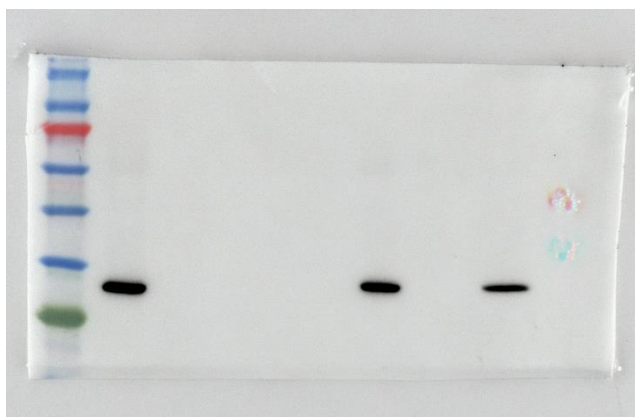

Supplement: Supplementary file 1 [file vetsci-13-00059-s001.zip › vetsci-4035912-supplementary.pdf]
